# Hepatitis B Core-Related Antigen as Surrogate Biomarker of Intrahepatic Hepatitis B Virus Covalently-Closed-Circular DNA in Patients with Chronic Hepatitis B: A Meta-Analysis

**DOI:** 10.3390/diagnostics11020187

**Published:** 2021-01-28

**Authors:** Gian Paolo Caviglia, Angelo Armandi, Chiara Rosso, Davide Giuseppe Ribaldone, Rinaldo Pellicano, Sharmila Fagoonee

**Affiliations:** 1 Department of Medical Sciences, University of Turin, 10124 Turin, Italy; angelo.armandi@unito.it (A.A.); chiara.rosso@unito.it (C.R.); davidegiuseppe.ribaldone@unito.it (D.G.R.); 2 Unit of Gastroenterology, Città della Salute e della Scienza di Torino-Molinette Hospital, 10126 Turin, Italy; rinaldo_pellican@hotmail.com; 3 Institute of Biostructure and Bioimaging (CNR), Molecular Biotechnology Center, 10126 Turin, Italy

**Keywords:** chronic HBV infection, HBcrAg, HBeAg, HBsAg, HBV cccDNA

## Abstract

Hepatitis B virus (HBV) covalently-closed-circular (ccc)DNA is the key molecule responsible for viral persistence within infected hepatocytes. The evaluation of HBV cccDNA is crucial for the management of patients with chronic HBV infection and for the personalization of treatment. However, the need for liver biopsy is the principal obstacle for the assessment of intrahepatic HBV cccDNA. In the last decade, several studies have investigated the performance of hepatitis B core-related antigen (HBcrAg) as a surrogate of HBV cccDNA amount in the liver. In this meta-analysis, we collected 14 studies (1271 patients) investigating the correlation between serum HBcrAg and intrahepatic HBV cccDNA. Serum HBcrAg showed a high correlation with intrahepatic HBV cccDNA (*r* = 0.641, 95% confidence interval (CI) 0.510–0.743, *p* < 0.001). In a head-to-head comparison, we observed that the performance of HBcrAg was significantly superior to that of hepatitis B surface antigen (*r* = 0.665 vs. *r* = 0.475, respectively, *p* < 0.001). Subgroup analysis showed that the correlation between HBcrAg and intrahepatic HBV cccDNA was high, both in hepatitis B e antigen-positive and -negative patients (*r* = 0.678, 95% CI 0.403–0.840, *p* < 0.001, and *r* = 0.578, 95% CI 0.344–0.744, *p* < 0.001, respectively). In conclusion, the measurement of serum HBcrAg qualifies as a reliable non-invasive surrogate for the assessment of an intrahepatic HBV cccDNA reservoir.

## 1. Introduction

Hepatitis B virus (HBV) infection is a major health problem worldwide [1]; the estimated prevalence of HBV infected patients in the world is approximately 257 million (3.7%) [2]. Chronic hepatitis B (CHB) is the result of an acute, unresolved infection that overtime may lead to cirrhosis and its complications such as liver failure and hepatocellular carcinoma (HCC) [3].

HBV covalently-closed-circular-(ccc)DNA is the key molecule responsible for the persistence of the virus within infected hepatocytes [4], even decades after resolution of HBV infection [5]. The HBV minichromosome acts as a template for all viral transcripts including the sub-genomic RNAs, pre-core RNA, and pre-genomic RNA [6].

Measuring the quantity and replication activity of HBV cccDNA is of paramount importance to improve the management of patients with CHB infection and to tailor individualized treatment strategies [7]. Unfortunately, the direct assessment of intrahepatic HBV cccDNA reservoir is limited in clinical practice by the need for liver biopsy [8,9]. Lately, exosomes derived from the serum of CHB patients were found to contain both HBV nucleic acids and HBV proteins, and could act as carriers of the virus, its nucleic acids, and proteins for further infection of uninfected hepatocytes [10]. Exosomes are 30–150 nm diameter bilipid-layered vesicles secreted by almost all cell types into body fluids including serum, plasma, saliva, and urine. Exosomes contain proteins, lipids, several RNA species as well as DNA, which can reflect the status of the host cells. Exosomes can participate in HBV replication and modulation of the host’s immune response, and their miRNA content can serve as biomarkers for HBV diagnosis [11]. Interestingly, the finding of HBV cccDNA inside exosomes by polymerase chain reaction (PCR) suggests that patient serum exosomes are promising sources of nucleic acids for HBV cccDNA analysis [10]. Further studies are needed in this field.

Among the available serum biomarkers, HBV DNA strongly correlates with intrahepatic HBV cccDNA levels; however, during antiviral treatment with nucleos(t)ide analogues (NAs), HBV DNA became rapidly undetectable and thus no longer informative [12]. Hepatitis B surface antigen (HBsAg), the serological hallmark of HBV infection, has been proposed to reflect the liver content of HBV cccDNA [13,14]. Recently, it has been shown that different mechanisms exist for HBsAg synthesis and secretion including transcription from HBV S-gene sequences integrated into the host genome [15]. Therefore, the reliability of HBsAg as a surrogate of intrahepatic HBV cccDNA may be questionable.

Hepatitis B core-related antigen (HBcrAg) is a composite biomarker that simultaneously measures hepatitis B core antigen (HBcAg), hepatitis B e antigen (HBeAg), and a 22 KDa core-related protein (p22cr) that constitutes the capsid of HBV empty particles [16]. This biomarker proved to be useful for the discrimination between the different phases of chronic HBV infection (particularly for the correct identification of patients with HBeAg-negative chronic infection) [17,18,19], for the management of patients under antiviral treatment [20], for the prediction of HBV reactivation following pharmacological immunosuppression [21], and for the stratification of the risk of HCC development as well as its recurrence [22]. Finally, HBcrAg exhibited a good correlation with intrahepatic HBV cccDNA quantity and productivity [23].

Here, we performed a meta-analysis on the value of HBcrAg as a surrogate of intrahepatic HBV cccDNA. Furthermore, we evaluated the performance of HBcrAg compared to HBsAg as indirect markers of HBV cccDNA, and we conducted a subgroup analysis according to HBeAg-positivity.

## 2. Materials and Methods

### 2.1. Search Strategy

This meta-analysis was performed according to the PRIMA (Preferred Reporting Items for Systematic Reviews and Meta-analyses) guidelines [24]. Original research articles published in English on the correlation between serum HBcrAg and intrahepatic HBV cccDNA quantity were identified through the PubMed (https://pubmed.ncbi.nlm.nih.gov) and Scopus (https://www.scopus.com) databases. The search strategy was based on the following terms: “(HBcrAg[All Fields] OR hepatitis B core-related antigen[All Fields]) AND (cccDNA[All Fields] OR covalently-closed-circular DNA[All Fields])”. The search on both databases was performed on 20 November 2020.

### 2.2. Study Selection

Two authors (G.P.C. and R.P.) independently reviewed the titles and the abstract of the studies retrieved from the electronic search and selected those potentially relevant for the purpose of the meta-analysis. The full-text versions of selected studies were assessed by three authors (G.P.C., R.P., and S.F.) to determine whether the inclusion criteria were satisfied.

The inclusion criteria were: (1) original research articles published in English; and (2) studies reporting the correlation coefficients between HBcrAg and intrahepatic HBV cccDNA. No restrictions were imposed concerning virologic and clinical features of patients included in the studies, method for HBV cccDNA measurement, and ongoing antiviral therapy. Exclusion criteria were: (1) studies that did not estimate the correlation between HBcrAg and intrahepatic HBV cccDNA; (2) in vitro studies; and (3) reviews, case reports, and meta-analysis.

### 2.3. Index and Reference Test

The measurement of serum HBcrAg was defined as the index test. Currently, serum HBcrAg can be evaluated only by a chemiluminescent enzyme immunoassay (CLEIA) on the Lumipulse^®^ *G* system (Fujirebio, Tokyo, Japan). The assay measures HBeAg, HBcAg, and p22cr and the concentration is calculated by comparison with a standard curve generated using recombinant pro-HBeAg. The immunoreactivity of pro-HBeAg at 10 fg/mL is defined as 1 U/mL. HBcrAg values are usually expressed as Log U/mL, with a measurement range between 3.0–7.0 Log U/mL. Some authors used to report HBcrAg concentration in serum as kU/mL.

The direct quantitation of intrahepatic HBV cccDNA was defined as the reference test (i.e., gold standard). However, it should be noted that no standard method has yet been identified for the measurement of HBV cccDNA; there is still no consensus on the protocol for HBV cccDNA isolation from liver tissue including the enzymatic digestion of relaxed HBV DNA, the specific primers for HBV cccDNA amplification, and the normalization of quantity PCR data. Intrahepatic HBV cccDNA is usually reported as copies/cell, copies/µg or copies/cell equivalent (cEq).

### 2.4. Data Extraction and Quality Assessment

From selected papers, the same two authors (G.P.C. and R.P.) extracted data regarding authors, country, year of publication, type of study, number of patients, virologic and clinical characteristics of patients, methods used for HBV cccDNA quantification, mean or median HBV cccDNA, HBcrAg, and HBsAg values, correlation coefficients (*r*), and *p* values.

The quality of included studies was assessed according to the QUADAS-2 (Quality Assessment of Diagnostic Accuracy Studies version 2) criteria [25].

### 2.5. Statistical Analysis

The meta-analysis was performed using MedCalc^®^ software version 18.9.1 (MedCalc bvba, Ostend, Belgium). The test for inter-rater agreement (Cohen Kappa statistics; *K*) was used to evaluate the agreement between investigators. The pooled correlation coefficient with 95% confidence interval (CI) was calculated from the number of cases, the *r* with 95% CI from all the included studies.

Forest plots showing the overall effect and funnel plots for publication bias assessment were constructed. According to the presence of heterogeneity, a fixed or random effects model was employed. Cochran’s *Q* and *I*^2^ statistics were used to detect heterogeneity; a *p* value < 0.05 was considered as indicative of heterogeneity.

Begg and Mazumdar’s rank correlation test (Kendall’s tau; *Τ*) was performed to measure the funnel plots’ asymmetry [26]. The standard normal deviate, defined as the natural logarithm of estimate divided by its standard error (SE), was correlated to the estimate’s precision, defined as the inverse of the SE. SE was calculated with the following formula: SE = (ln UB − ln LB)/2 × 1.96, where UB and LB are the upper and lower bound of the 95% CI of *r*, respectively.

Comparison between correlation coefficients was performed by z-statistics.

## 3. Results

### 3.1. Description of the Study Population

**Figure 1:**
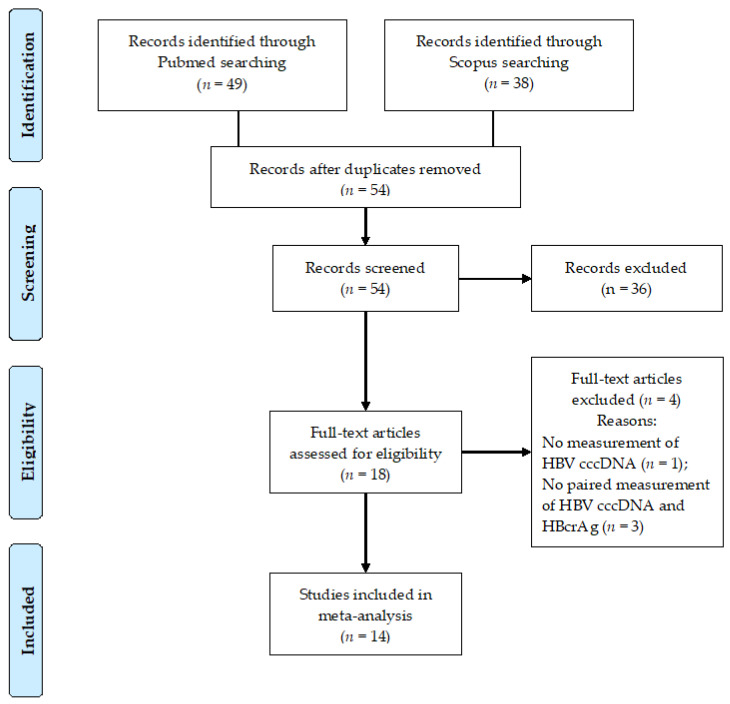
Flow diagram of the study. Abbreviations: covalently-closed-circular DNA (cccDNA), hepatitis B core-related antigen (HBcrAg), hepatitis B virus (HBV), number (*n*).

Of the 54 articles identified through our systematic search, 14 met the eligibility criteria (Figure 1) [23,24,25,26,27,28,29,30,31,32,33,34,35,36,37,38,39]. There was no disagreement among authors regarding the eligibility of original articles finally included in the meta-analysis (*K* statistics = 1).

Overall, 1271 patients chronically infected with HBV were included in the present meta-analysis (Table 1). Most studies were performed in East Asia (11/14; 78.6%) and the majority in China (*n* = 6) [27,31,32,34,36,37]. Only three studies came from European countries (two from France and one from Italy) [23,38,39]. Consistently, the most represented genotypes were the HBV genotype C (*n* = 376), followed by genotype B (*n* = 230); the HBV genotype was not available for 525 patients (41.3%). Data concerning HBeAg-positivity was available for 985 patients (77.5%); 515 patients were HBeAg-positive (52.3%), while 470 were HBeAg-negative (47.7%). The liver disease was complicated by HCC in 266 patients (20.9%) [26,31,32]; 31 patients were co-infected with human immunodeficiency virus (HIV) [36]. Only the minority of patients were under nucleos(t)ide analogues (NAs) treatment at the time of HBcrAg and HBV cccDNA evaluation [29,39]. Two studies assessed serum HBV biomarkers and intrahepatic HBV cccDNA before NAs initiation and during treatment [31,36].

In most studies, HBV cccDNA was isolated from frozen liver biopsy/specimens stored at −80 °C; only Chen and colleagues isolated HBV cccDNA from formalin-fixed paraffin-embedded liver tissue [32,37]. In order to achieve a HBV cccDNA specific quantitation, authors used specific primers for HBV cccDNA amplification (i.e., primers flanking the gap region of HBV genome), with [23,31,32,34,36,37,38,39] or without [28,29,30,33,35] previous plasmid-safe ATP dependent DNase treatment to digest single-strand and relaxed double-strand DNA isolated from liver tissue samples. In one study, intrahepatic HBV DNA was purified by the modified Hirt procedure and amplified by the Invader HBV assay [27]. HBV cccDNA was quantified by real-time PCR in the majority of the studies; in one study, HBV cccDNA was quantified by droplet-digital PCR [38].

**Figure 2:**
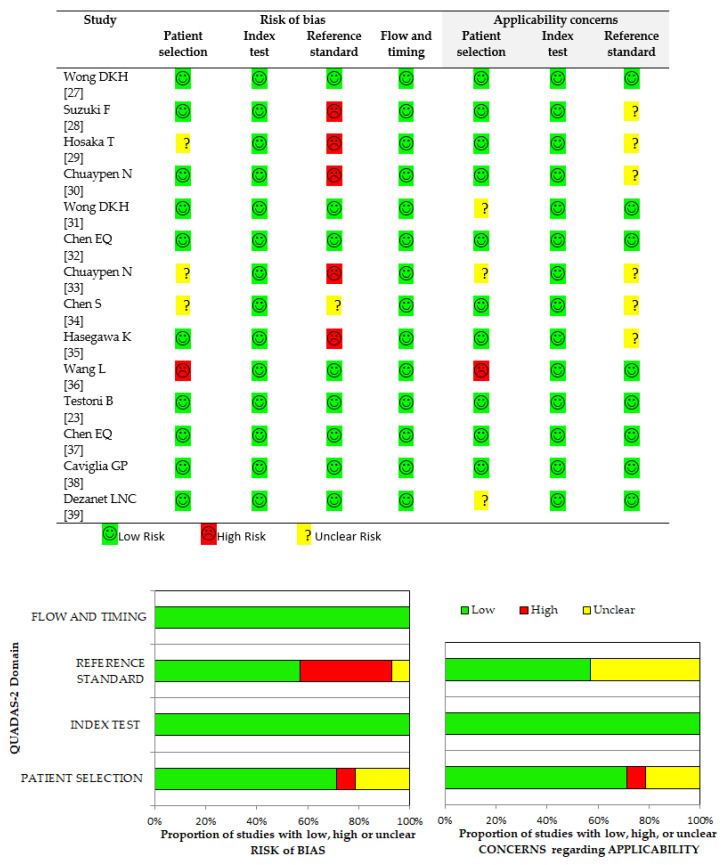
Overall methodology quality assessment of included studies using QUADAS-2 criteria. Abbreviations: Quality Assessment of Diagnostic Accuracy Studies version 2 (QUADAS-2).

The methodological quality assessment of the included research articles is shown in Figure 2. The overall quality of the studies was high. To note, the higher rate of a potential source of bias pertained to reference standard domain; five out of 14 studies (35.7%) reported no enzymatic digestion of total intrahepatic DNA prior to HBV cccDNA amplification. Indeed, this methodological aspect is crucial in order to improve PCR specificity [37].

### 3.2. Correlation between Serum HBcrAg and Intrahepatic HBV cccDNA

**Figure 3:**
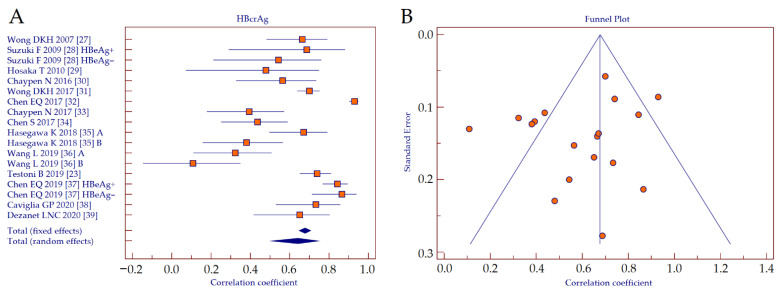
Forest plot (**A**) and funnel plot (**B**) of the correlation between serum HBcrAg and intrahepatic HBV cccDNA. Abbreviations: hepatitis B core-related antigen (HBcrAg), hepatitis B virus (HBV), covalently-closed-circular DNA (cccDNA).

The correlation coefficients retrieved from correlation analyses between serum HBcrAg values and intrahepatic HBV cccDNA were analyzed by using forest plot (Figure 3A). Since the studies showed heterogeneity (Cochran’s *Q*, *p* < 0.001; *I*^2^ = 91.6%), a random effects model was applied. The result showed a strong positive correlation between HBcrAg and HBV cccDNA (*r* = 0.641, 95% CI 0.510–0.743, *p* < 0.001). A funnel plot was depicted to visually inspect for possible publication bias (Figure 3B). Kendall rank correlation analysis showed significant publication bias (*T* = −0.503, *p* = 0.003).

### 3.3. Comparison between HBcrAg and HBsAg Performance

**Figure 4:**
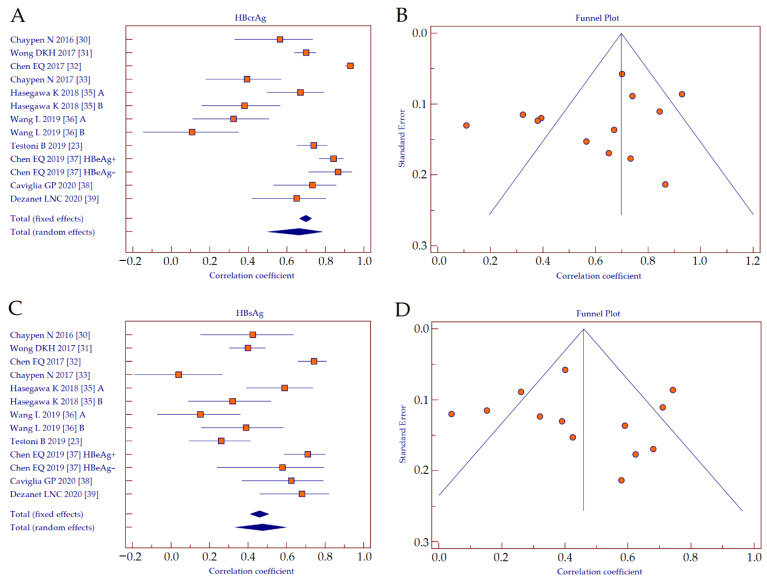
Forest plot (**A**) and funnel plot (**B**) of the correlation between serum HBcrAg and intrahepatic HBV cccDNA, and forest plot (**C**) and funnel plot (**D**) of the correlation between serum HBsAg and intrahepatic HBV cccDNA. Abbreviations: hepatitis B core-related antigen (HBcrAg), hepatitis B surface antigen (HBsAg), hepatitis B virus (HBV), covalently-closed-circular DNA (cccDNA).

We performed a head-to-head comparison of the performance of HBcrAg and HBsAg as surrogate biomarkers of intrahepatic HBV cccDNA including only the studies that provided both data [23,27,28,29,30,32,33,34,35,36]. The correlation coefficients retrieved from correlation analyses between serum HBcrAg values and intrahepatic HBV cccDNA were analyzed by using forest plot (Figure 4A). Since the studies showed heterogeneity (Cochran’s *Q*, *p* < 0.001; *I*^2^ = 93.6%), a random effects model was applied. The result showed a strong positive correlation between HBcrAg and HBV cccDNA (*r* = 0.665, 95% CI 0.507–0.779, *p* < 0.001). A funnel plot was depicted to visually inspect for possible publication bias (Figure 4B). Kendall rank correlation analysis showed significant publication bias (*T* = −0.436, *p* = 0.033).

The correlation coefficients retrieved from correlation analyses between serum HBsAg values and intrahepatic HBV cccDNA were analyzed by using forest plot (Figure 4C). Since the studies showed heterogeneity (Cochran’s *Q*, *p* < 0.001; *I^2^* = 85.6%), a random effects model was applied. The result showed a moderate positive correlation between HBsAg and HBV cccDNA (*r* = 0.475, 95% CI 0.339–0.592, *p* < 0.001). The corresponding funnel plot is depicted in Figure 4D. Kendall rank correlation analysis showed significant publication bias (*T* = 0.821, *p* < 0.001).

By direct comparison of the summary correlation coefficients, we observed that the performance of HBcrAg was significantly superior to HBsAg (z-statistics, *p* < 0.001).

### 3.4. Performance of HBcrAg According to HBeAg-Positivity

**Figure 5:**
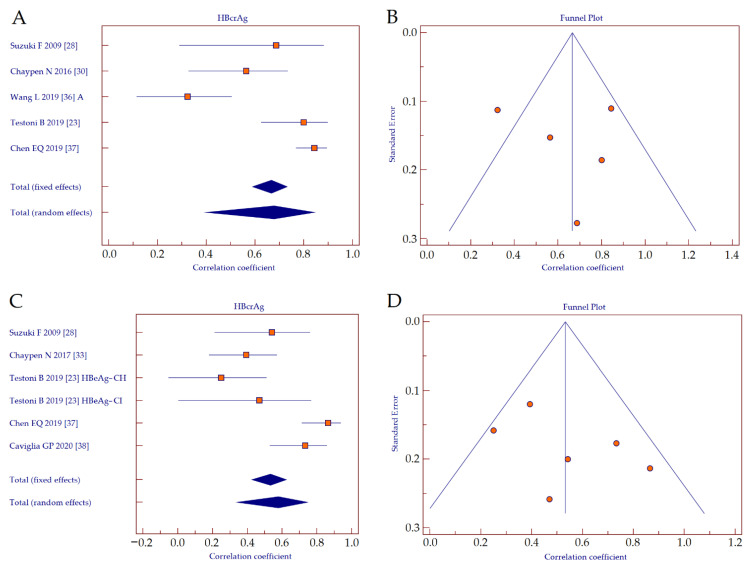
Forest plot (**A**) and funnel plot (**B**) of the correlation between serum HBcrAg and intrahepatic HBV cccDNA in HBeAg-positive patients, and forest plot (**C**) and funnel plot (**D**) of the correlation between serum HBcrAg and intrahepatic HBV cccDNA in HBeAg-negative patients. Abbreviations: hepatitis B core-related antigen (HBcrAg), hepatitis B e antigen (HBeAg), hepatitis B virus (HBV), covalently-closed-circular DNA (cccDNA).

We performed a sub-analysis to investigate the performance of HBcrAg as a surrogate biomarker of intrahepatic HBV cccDNA, according to HBeAg-positivity. The correlation coefficients retrieved from studies investigating the between serum HBcrAg values and intrahepatic HBV cccDNA in HBeAg-positive patients were analyzed by using a forest plot (Figure 5A) [23,25,27,33,34]. Since the studies showed heterogeneity (Cochran’s *Q*, *p* < 0.001; *I*^2^ = 88.9%), a random effects model was applied. The result showed a strong positive correlation between HBcrAg and HBV cccDNA (*r* = 0.678, 95% CI 0.403–0.840, *p* < 0.001). A funnel plot was depicted to visually inspect for possible publication bias (Figure 5B). Kendall rank correlation analysis indicated no publication bias (*T* = −0.200, *p* = 0.462).

The correlation coefficients retrieved from studies investigating the between serum HBcrAg values and intrahepatic HBV cccDNA in HBeAg-negative patients were analyzed by using forest plot (Figure 5C) [23,25,30,34,35]. Since the studies showed heterogeneity (Cochran’s *Q*, *p* < 0.001; *I*^2^ = 77.3%), a random effects model was applied. The result showed a moderate positive correlation between HBcrAg and HBV cccDNA (*r* = 0.578, 95% CI 0.344–0.744, *p* < 0.001). The corresponding funnel plot is depicted in Figure 5D. Kendall rank correlation analysis indicated no publication bias (*T* = −0.467, *p* = 0.133).

By direct comparison of the summary correlation coefficients, we observed no significant differences concerning the performance of HBcrAg between HBeAg-positive and HBeAg-negative patients (z-statistics, *p* = 0.069).

## 4. Discussion

Different findings of the present meta-analysis are noteworthy. First, the measurement of serum HBcrAg proved to be a reliable non-invasive surrogate of the quantity of intrahepatic HBV cccDNA, despite an overall population of 1271 patients with different demographic, virologic, and clinical characteristics. Second, in a head-to head-comparison, HBcrAg showed a significantly higher performance compared to serum HBsAg. Finally, serum HBcrAg showed a good correlation to intrahepatic HBV cccDNA reservoir, irrespective of HBeAg-positivity.

Although no standardized method for HBV cccDNA assessment is currently available, major efforts have been done in the last decades to design sensitive and specific molecular assays for the investigation of the different replicative DNA intermediates of HBV. To improve specificity, it has been shown that treatment of liver DNA extracts with enzymatic digestion prior to PCR amplification is mandatory [40]. However, which could be the more appropriate protocol is still a matter of debate [6,41,42]. On this premise, the possibility to accurately infer the amount of HBV cccDNA in the liver by using a standardized, non-invasive, and accurate tool is of considerable clinical relevance. Indeed, novel therapeutic options for the treatment of patients with CHB are under investigation [43]. Standard antiviral treatment with NAs can inhibit viral replication, but is not curative and must be administered life-long because it is ineffective against the HBV cccDNA [44]. New therapeutic approaches include strategies to prevent HBV cccDNA synthesis, to enhance its degradation, and to silence its transcription [45]. Therefore, it will be crucial to monitor in a non-invasive but accurate manner the modifications to intrahepatic HBV cccDNA quantity induced by therapy [46,47]. In this regard, serum HBcrAg qualifies as a suitable tool to be implemented in clinical practice in order to improve the patients’ management.

Different biomarkers have been proposed as a surrogate of the intrahepatic HBV cccDNA pool. Here, we performed a direct comparison between HBcrAg and HBsAg performance and observed that the former more strongly correlated with HBV cccDNA compared to the latter (*r* = 0.665 vs. *r* = 0.475, respectively). As a matter of fact, integrated HBV DNA into the host genome can contribute significantly to the serum expression of HBsAg [48], while HBcAg and HBeAg are translated from the pre-genomic (pg) and pre-core HBV RNAs, that originate solely from the HBV cccDNA [49]. Similarly, the p22cr protein, which contains an uncleaved signal sequence and lacks a C-terminal arginine-rich domain, derives from the pre-core HBV RNA [50]. Furthermore, compared to serum HBsAg [51], recent evidence suggests that HBcrAg serum values are not affected by HBV genotype [52]. Taken together, these results further corroborate the reliability of HBcrAg as an indirect biomarker of intrahepatic HBV cccDNA.

Finally, we performed a sub-analysis to assess the performance of HBcrAg according to HBeAg-positivity. The overall correlation observed between serum HBcrAg and intrahepatic HBV cccDNA tended to be higher in the HBeAg-positive compared to HBeAg-negative patients, despite the fact that a statistical significance was not reached. Interestingly, no risk of publication bias was evinced, either by funnel plot evaluation or by Begg and Mazumdar’s correlation analysis. Conversely, a high risk of publication bias emerged from the whole analysis of the 14 studies included in the meta-analysis. Likely, among the different characteristics of the 1271 analyzed patients, serum HBeAg-positivity had a significant impact on HBcrAg performance. Consistently, it has been shown that the major variance of HBcrAg can be attributed to HBeAg in patients with HBeAg-positive chronic infection (i.e., immune-tolerant) and HBeAg-positive chronic hepatitis (i.e., immune-clearance), while HBV DNA is a major determinant of HBcrAg in patients with HBeAg-negative chronic hepatitis [53].

The present meta-analysis has some limitations that deserve to be discussed. As above-mentioned, the overall population included consisted of 1271 patients, mostly of Asiatic ethnicity and predominantly infected with HBV genotypes B and C. Despite three studies assessing HBcrAg performance in Caucasian patients [23,38,39] showing similar results, the data should be interpreted with caution when dealing with patients of ethnicity different from Asian and chronically infected with HBV genotype non-B/C. Another important limitation is that we assessed the value of HBcrAg as an indirect biomarker of the intrahepatic HBV cccDNA amount, but we did not investigate the value of HBcrAg as a surrogate of HBV cccDNA transcriptional activity. To date, few studies have investigated such relations, which are not enough to perform a substantial analysis; however, a recent study showed that among serum HBV biomarkers, only HBcrAg was correlated to the pgRNA/cccDNA ratio in HBeAg-negative patients [23]. Further studies are needed to deepen this aspect, also taking into consideration the promising results from studies investigating the value of novel potential biomarkers such as circulating HBV RNA [54,55]. Eventually, it would be interesting to evaluate the presence of HBcrAg within exosomes, which are now widely studied for their biomarker and diagnostic potential.

## 5. Conclusions

In conclusion, the measurement of serum HBcrAg represents a reliable non-invasive tool for the indirect assessment of intrahepatic HBV cccDNA. The performance of HBcrAg as a surrogate of HBV cccDNA quantity was significantly superior to quantitative HBsAg; furthermore, HBcrAg exhibited a strong correlation with the HBV cccDNA pool in both HBeAg-positive and HBeAg-negative patients. To date, the measurement of HBcrAg may represent an appropriate tool to help clinicians in the management of patients chronically infected with HBV.

## Figures and Tables

**Table 1:** Characteristics of the studies included in the meta-analysis.

Study	Country	Year	Patients (*n*)	HBeAg (+/−)	HBV Genotype	Therapy	HBcrAg	HBsAg	HBV cccDNA	*r* * p-Value	*r* ** p-Value
Wong DKA [27]	China	2007	54	17/37	n.a.	No	1180 (<1.0–9.0 × 10^5^) kU/mL	n.a.	1.3 (<0.002–23.3) copies/cell	*r* = 0.664 *p* < 0.001	n.a.
Suzuki F [28]	Japan	2009	44	16/28	n.a.	No	5.05 ± 1.62 Log U/mL e+: 6.53 ± 1.14 Log U/mL e−: 4.20 ± 1.18 Log U/mL	n.a. n.a. n.a.	4.46 ± 0.87 Log copies/mg e+: 4.88 ± 1.06 Log copies/mg e−: 4.23 ± 0.64 Log copies/mg	n.a. *r* = 0.687 *p* = 0.003 *r* = 0.542 *p* = 0.003	n.a. n.a. n.a.
Hosaka T [29]	Japan	2010	55 ^A^	23/32	C = 51 Other = 9	30 LAM 17 LAM + ADV 8 ETV	5.0 (<3.0–> 6.8) Log U/mL	n.a.	4.2 (3.0–5.0) Log copies/µg ^B^	*r* = 0.479 *p* = 0.028 ^B^	n.a.
Chuaypen N [30]	Thailand	2016	46	46/0	B = 5 C = 41	No	8.1 (7.7–8.4) Log U/mL	3.9 (3.7–4.1) Log IU/mL	1.6 (1.2–1.9) copies/cEq	*r* = 0.564 *p* = 0.001	*r* = 0.424 *p* = 0.020
Wong DKA [31]	China	2017	138	77/61	B = 40 C = 82 n.a. = 16	Baseline 1 year NAs 6–12 years NAs	586 (1–1.1 × 10^7^) kU/mL ^C^	3.3 (−1.3–5.9) Log IU/mL ^C^	1.1 (0.005–258) copies/cell ^C^	*r* = 0.70 *p* < 0.001 ^C^	*r* = 0.40 *p* < 0.001 ^C^
Chen EQ [32]	China	2017	139	111/28	B = 88 C = 51	No	9.23 ± 2.86 Log U/mL	4.15 ± 0.86 Log IU/mL	7.33 ± 1.03 Log copies/10^6^ cells	*r* = 0.929 *p* < 0.001	*r* = 0.742 *p* < 0.001
Chuaypen N [33]	Thailand	2018	121 ^D^	0/121	B = 25 C = 96	No	R: 4.1 ± 1.3 Log U/mL NR: 4.4 ± 1.1 Log U/mL	R: 3.2 ± 0.4 Log IU/mL NR: 3.6 ± 0.5 Log IU/mL	R: 0.4 ± 0.9 Log copies/cEq NR: 0.4 ± 1.2 Log copies/cEq	*r* = 0.393 *p* = 0.009	*r* = 0.040 *p* = 0.737
Chen S [34]	China	2018	160 ^E^	n.a.	n.a.	n.a.	5.10 (1.96–8.50) Log U/mL	250 (0.14–250) IU/mL	n.a.	*r* = 0.436 *p* < 0.001 ^F^	n.a.
Hasegawa K [35]	Japan	2018	126 ^G^	n.a.	n.a.	Untreated, *n* = 57 (A) previous NAs, *n* = 69 (B)	A: 3.0 (2.0–7.0) Log U/mL B: 4.1 (2.0–7.0) Log U/mL	A: 1692.7 (0.05–91960.7) IU/mL B: 1050.0 (0.05–71583.0) IU/mL	A: 3.0 (1.5–5.8) Log copies/µg B: 3.4 (1.7–4.8) Log copies/µg	A: *r* = 0.67 *p* < 0.001 B: *r* = 0.38 *p* = 0.007	A: *r* = 0.59 *p* < 0.001 B: *r* = 0.32 *p* = 0.007
Wang L [36]	China	2019	82	82/0 (A); 44/12 (B)	n.a.	Baseline (A) 2 years NAs (B)	A: 7.97 ± 0.96 Log U/mL B: 5.74 ± 1.10 Log U/mL	A: 4.05 ± 0.64 Log IU/mL B: 3.32 ± 0.90 Log IU/mL	A: 0.67 ± 0.74 Log copies/cell B: −0.94 ± 0.60 Log copies/cell	A: *r* = 0.323 ^H^ *p* < 0.001 B: *r* = 0.108 *p* = 0.403	A: *r* = 0.152 *p* = 0.172 B: *r* = 0.39 *p* = 0.002
Testoni B [23]	France	2019	130	36/94	A = 20 B = 4 C = 13 D = 51 E = 14 F = 3	No	5.3 (4.0–7.6) Log U/mL e+: 8 (7.3–8.3) Log U/mL e−: 4.0 (3.7–4.9) Log U/mL	3.9 (3.4–4.3) Log IU/mL e+: 4.61 (4.1–5.2) Log IU/mL e−: 3.74 (3.2–4.1) Log IU/mL	0.15 (0.06–1.34) copies/cell e+: 6.3 (1.4–18.1) copies/cell e−: 0.09 (0.03–0.2) copies/cell	*r* = 0.74 *p* <0.001 ^I^ e+: *r* = 0.80 *p* <0.001 ^L^ e− CH: *r* = 0.25 *p* = n.s. ^I^ e− CI: *r* = 0.47 *p* = 0.05 ^I^	*r* = 0.26 *p* = 0.044 ^I^ e+: *r* = 0.33 *p* = 0.01 ^L^ e− CH: *r* = −0.4 *p* = 0.01 ^I^ e− CI: *r* = −0.03 *p* = n.s. ^I^
Chen EQ [37]	China	2019	110	85/25	B = 68 C = 42	No	e+: 10.30 (6.00–12.30) Log U/mL e−: 5.40 (3.28–7.20) Log U/mL	e+: 4.59 (0.82–5.10) Log IU/mL e−: 3.49 (0.99–4.01) Log IU/mL	e+: 7.46 (5.11–8.17) Log copies/10^6^ cells e−: 6.03 (5.00–6.85) Log copies/10^6^ cells	e+: *r* = 0.843 *p* < 0.001 e−: *r* = 0.865 *p* < 0.001	e+: *r* = 0.710 *p* < 0.001 e−: *r* = 0.579 *p* = 0.002
Caviglia GP [38]	Italy	2020	35	0/35	D = 35	No	3.8 ± 1.8 Log U/mL	3.13 ± 1.31 Log IU/mL	3.11 ± 1.14 Log copies/10^5^ cells	*r* = 0.733 *p* < 0.001	*r* = 0.624 *p* < 0.001
Dezanet LNC [39]	France	2020	31 ^M^	22/9	A = 11 D = 1 E = 1 G = 4 n.a. = 14	NAs + ART (*n* = 22)	5.5 (3.1–7.0) Log U/mL	4.0 (3.2–4.5) Log IU/mL	0.26 (0.0–2.89) copies/cell	*r* = 0.65 *p* < 0.001 e+: *r* = 0.40 *p* = 0.07 e−: *r* = 0.22 *p* = 0.5	*r* = 0.68 *p* < 0.001 e+: *r* = 0.42 *p* = 0.10 e−: *r* = 0.68 *p* = 0.03

* Correlation between serum HBcrAg and intrahepatic HBV cccDNA values. ** Correlation between serum HBsAg and intrahepatic HBV cccDNA values. ^A^ Patients with HCC. ^B^ HBV cccDNA was measured in liver specimens from 22 out of 55 patients. ^C^ The analysis was performed of 305 samples from 138 patients: 138 pre-treatment samples, 124 after one year of receiving NAs and 43 after 6–12 years of therapy. ^D^ The original study reported the mean baseline HBcrAg, HBsAg, and HBV cccDNA values according to virologic response to Peg-IFN alone or combined to ETV (R vs. NR). ^E^ Patients with HCC. ^F^ HBV cccDNA was measured in liver specimens from 89 out of 160 patients. ^G^ The study cohort included 51 (40.5%) patients with HCC. ^H^ Three patients were excluded from correlation analysis. ^I^ Only patients with HBcrAg > 3 Log U/mL were included in the analysis. ^L^ Correlation analysis was performed only on the 32 patients with HBeAg-positive chronic hepatitis (the 4 patients with HBeAg-positive chronic infection were excluded). ^M^ 38 liver biopsies from 31 HIV–HBV coinfected patients. Abbreviations: adefovir (ADV), antiretroviral therapy (ART), cell equivalent (cEq), chronic infection (CI), chronic hepatitis (CH), correlation coefficient (*r*), covalently-closed-circular DNA (cccDNA), e antigen-positive (e+), e antigen-negative (e−), entecavir (ETV), hepatitis B core-related antigen (HBcrAg), hepatitis B e antigen (HBeAg), hepatitis B surface antigen (HBsAg), hepatitis B virus (HBV), hepatocellular carcinoma (HCC), human immunodeficiency virus (HIV), lamivudine (LAM), non-responder (NR), not available (n.a.), nucleos(t)ide analogues (NAs), number (*n*), responder (R).
